# Butylidenephthalide antagonizes cromakalim-induced systolic pressure reduction in conscious normotensive rats

**DOI:** 10.1186/s12906-015-0877-z

**Published:** 2015-10-05

**Authors:** Chung-Hung Shih, Yu-Jing Lin, Chi-Ming Chen, Wun-Chang Ko

**Affiliations:** Department of Internal Medicine, Taipei Medical University Hospital, 252 Wu-Hsing St., Taipei, 110 Taiwan; Department of Pharmacology, College of Medicine, Taipei Medical University, 250 Wu-Hsing St., Taipei, 110 Taiwan; Department of Medicinal Chemistry, College of Pharmacy, Taipei Medical University, 250 Wu-Hsing St., Taipei, 110 Taiwan

**Keywords:** 4-Aminopiridine, ATP-sensitive K^+^ channels, K_v_1 family of K^+^ channels, Butylidenephthalide, Conscious normotensive rats, Cromakalim

## Abstract

**Background:**

Butylidenephthalide (Bdph), a main constituent of *Ligusticum chuanxiong* Hort., was reported to have selective antianginal effect without changing blood pressure in conscious rat. Recently, we have observed that Bdph antagonized cromakalim, an ATP-dependent K^+^ channel opener, in guinea-pig trachea. Thus, we were interested in investigating whether Bdph at the dose without changing blood pressure antagonized cromakalim-induced systolic pressure reduction in conscious rats.

**Methods:**

Systolic arterial pressures of conscious rats were determined by using the indirect tail-cuff method.

**Results:**

Bdph (30 mg/kg, i.p.) did not affect baseline systolic pressure in conscious normotensive and spontaneous hypertensive rats. Bdph (30 mg/kg, i.p.) also did not affect log dose–response curves of prazosin, clonidine and Bay K 8644, a Ca^2+^ channel activator, in normotensive rats. However, Bdph (30 mg/kg, i.p.) similar to 4-aminopyridine (4-AP, 0.4 mg/kg, i.p.), a K^+^ channel blocker, non-parallelly but surmountably, and partially similar to glibenclamide (GBC, 10 mg/kg, i.v.), an ATP-sensitive K^+^ channel blocker, surmountably but not parallelly rightward shifted the log dose-systolic pressure reduction curve of cromakalim, an ATP-sensitive K^+^ channel opener, in normotensive rats, respectively.

**Discussion:**

The antagonistic effect of Bdph against cromakalim was similar to that of 4-AP, a K+ channel blocker of Kv1 family, and partially similar to that of GBC, an ATP-sensitive K+ channel blocker. Thus, Bdph may be a kind of K+ channel blockers, which have been reviewed to have a potential clinical use for Alzheimer disease. Indeed, Bdph has also been reported to reverse the deficits of inhibitory avoidance performance and improve memory in rats. Recently, 4-AP was reported to treat Episodic ataxia type 2 (EA2) which is a form of hereditary neurological disorder. Consistently, Bdph was recently reported to have antihyperglycemic activity in mice, since GBC is a powerful oral hypoglycemic drug.

**Conclusions:**

Bdph similar to 4-AP and partially similar to GBC may block K_v_1 family and ATP-sensitive K^+^ channels in conscious normotensive rats.

## Background

The rhizome of *Ligusticum chuanxiong* Hort. (previously named as *L. wallichii* Franch., Umbelliferae) have been used by the Chinese for several thousand years. In ancient medical literature, such as Shen-Nung-Pen-Tsao-Ching, the rhizome of *L. chuanxiong* Hort. was delineated to prevent and restore stroke-induced dyskinesia. From the neutral oil of the rhizome we isolated and purified three antispasmodics, butylidenephthalide (Bdph), ligustilide and butylphthalide [1, 2]. Bdph was reported to inhibit cyclooxygenase and to have anti-platelet effects [3]. Shimotsu-to, a prescription of traditional Chinese medicine, had antiproliferative effects in primary cultures of mouse aorta smooth muscle cells [4], mainly due to the effect of Bdph [5]. Both anti-platelet and antiproliferative effects of Bdph may benefit to prevent ischemic stroke. To recover from stroke-induced dyskinesia the damaged nervous tissue needs to repair by itself. The vasodilating effects of Bdph [6–8] improve the circulation and may partially benefit this restoration. Recently, Bdph was reported to provide neuroprotection by reducing the release of various proinflammatory molecules from activated microglia [9], Bdph was also reported to maintain stem cell pluripotency by activating the Jak2/Stat3 pathway and to increase the efficiency of induced pluripotent stem cells generation [10]. These results highlight the ability for these crude drugs to aid in the recovery from dyskinesia. Interestingly, Bdph was also reported to inhibit growth of hepatocellular carcinoma [11], colon cancer [12], and prostate cancer [13] with a high therapeutic ratio [14].

Bdph was reported to inhibit Ca^2+^ influx and cause relaxation in guinea-pig ileum [15], rat aortic ring [8] and rabbit aortic strip [6]. Bdph was also reported to increase flow in isolated guinea-pig heart [16] and rabbit ear [6]. However, Bdph was reported to have selective antianginal effect without changing blood pressure in conscious rat [7]. The effect of Bdph on blood pressure is obviously different from that of nitroglycerin, an antianginal drug, which occasionally causes hypotension and vertigo. Recently, we have reported that Bdph antagonized cromakalim, an ATP-sensitive K^+^ channel opener, in guinea-ping trachea [17]. Thus, we were interested in investigating whether Bdph at the dose without changing blood pressure antagonized cromakalim in conscious rat. In the preliminary test, an intraperitoneal (i.p.) injection of Bdph at 30 mg/kg did not influence the systolic pressure of conscious normotensive and spontaneous hypertensive rats. However, Bdph at 60 mg/kg (i.p.) induced systolic pressure reduction itself, and at 15 mg/kg (i.p.) did not antagonize the effect of cromakalim.

## Methods

### Drugs and animals

**Fig. 1:**
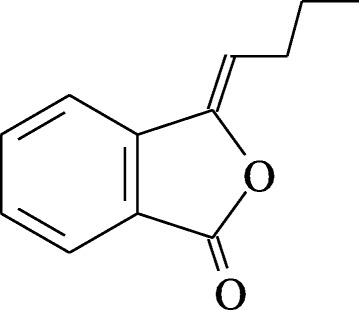
The chemical structure of butylidenephthalide (Bdph, mol. wt. 188.23)

Bdph was synthesized according to the previously described method [18]. The compound was light yellow oily substance. The structure is shown as Fig. 1. 4-Aminopyridine (4-AP, a K^+^ channel blocker) and glibenclamide (GBC, an ATP-sensitive K^+^ channel blocker [19]) were purchased from Sigma-Aldrich, St. Louis, MO, U.S.A. Prazosin (an α_1_-adrenoceptor antagonist), clonidine (an α_2_-adrenoceptor agonist), Bay K 8644 (a Ca^2+^ channel activator), and cromakalim (BRL 34915, an ATP-sensitive K^+^ channel opener), were gifts from Pfizer, U.S.A., Boehringer Sohn, Germany, Bayer Leverkusen, Germany, and SmithKline Beecham Pharmaceutical, U.K., respectively. Bdph and prazosin were dissolved in 95 % ethyl alcohol. When used, Bdph was diluted to 3 % in saline. Bay K 8644 and nifedipine were dissolved in a mixture of 2 % ethyl alcohol, 5 % polyethylene glycol 400 (PEG 400) and 1 % Tween 80 in saline. Cromakalim was dissolved in a mixture of PGE 400: saline (1:1, w/v). GBC was dissolved in 1 M NaOH first and then diluted in 5 % dextrose (1: 39). Clonidine and 4-AP were dissolved in saline.

Male normotensive (Wistar) and spontaneous hypertensive (SHR) rats, weighting 200 ~ 300 g, were purchased from the Animal Center of the Ministry of Science and Technology, Taipei, Taiwan. The animals were housed in ordinary cages at 22 ± 1 °C with a humidity of 50 ~ 60 % under a constant 12/12-h light/dark cycle and provided with food and water *ad libitum*. Under a protocol approved by the Animal Care and Use Committee of Taipei Medical University, the following *in vivo* experiments were performed.

### Determination of systolic arterial pressure

After training for a week, these conscious rats were individually put into acrylic restraining cage on a warm plate (38 ~ 40 °C), and kept their tails warm (35 ~ 37 °C). The systolic arterial pressure of rat was determined by using the indirect tail-cuff method [20]. Briefly, when the cuff pressure was elevated to cut off the blood flow by inflating a bulb, no pulse was detected by a pneumatic sensor (International Biomedical Inc., Houston, TX, U.S.A.), which was put along the tail artery of rat. When cuff pressure was reduced (−20 mmHg/s) to allow the blood flow, the pulse was detected by the sensor. As the beginning of pulse appeared the cuff pressure was the systolic pressure of rat.

### Effects of Bdph on systolic pressure in normotensive and spontaneous hypertensive rats

Systolic pressures of rats were determined in an interval of 15 min. Four baseline values were taken before Bdph (30 mg/kg, i.p.) or its vehicle (1 ml/kg, i.p., control) injection. After injection (0 min), both rats were continuously determined each 15 min for 1 h. After 1 h, however, normotensive rats were continuously determined each hr for another 2 h.

### Effect of Bdph on prazosin- or clonidine-induced systolic pressure reduction in normotensive rats

Systolic pressures of rats were determined in an interval of 15 min, with the exception that an additional determination was performed at 7.5 min after intravenous (i.v.) injection of clonidine to avoid losing effective values. Baseline values were taken before injection of Bdph (30 mg/kg, i.p.) or its vehicle. Thirty min after injection, prazosin (0.1 ~ 3 mg/kg, i.p.) or clonidine (0.003 ~ 0.03 mg/kg, i.v.) was additionally injected. In 1 h after injection of prazosin or clonidine, the maximal systolic pressure reduction was collected, and the log dose–response curve was constructed.

### Effect of Bdph on Bay K 8644-induced systolic pressure elevation in normotensive rats

Systolic pressures of rats were determined in an interval of 15 min, with the exception that an additional determination was performed at 7.5 min after intravenous (i.v.) injection of Bay K 8644 to avoid losing effective values. Two baseline values were taken before injection of Bdph (30 mg/kg, i.p.), nifedipine (1 mg/kg, i.v.), a reference drug, or their vehicles. Thirty min after injection of Bdph or 5 min after injection of nifedipine, Bay K 8644 (0.03 ~ 0.6 mg/kg, i.v.) was additionally injected. In 1 h after injection of Bay K 8644, the maximal hypertensive response was collected, and the log dose–response curve was constructed.

### Effect of Bdph on the responses of cromakalim in normotensive rats

Systolic pressures of rats were determined in an interval of 15 min, with the exception that an additional determination was performed at 7.5 min after i.v. injection of cromakalim to avoid losing effective values. Two baseline values were taken before injection of Bdph (30 mg/kg, i.p.), 4-Ap (0.4 mg/kg, i.p.), GBC (10 mg/kg, i.v.), or their vehicles. 4-AP and GBC were used as reference drugs. Thirty min after injection of Bdph, 4-AP or GBC, cromakalim was additionally injected (i.v.) at doses from 0.075 to 1.2 mg/kg for Bdph-, to 0.6 mg/kg for 4-AP-, and to 2.4 mg/kg for GBC-treated rats. In 1 h after injection of cromakalim, the maximal systolic pressure reduction was collected, and the log dose–response curve was constructed.

### Statistical analysis

The values of maximal response (E_max_) and slope (S) of these log dose–response curves were generated from the softward of SigmaPlot 10. The effective dose at 50 % of the maximal response (ED_50_) value was calculated from linear regression. All values were expressed as mean ± SEM, n was the number of experiment. Differences among three values were statistically calculated by one-way analysis of variance (ANOVA), and then determined by Dunnett’s test. The difference between two values was determined by Student's paired or unpaired *t*-test. Differences of *P* < 0.05 were considered statistically significant.

## Results

### No effect of Bdph on systolic pressure in normotensive or spontaneous hypertensive rats

**Fig. 2:**
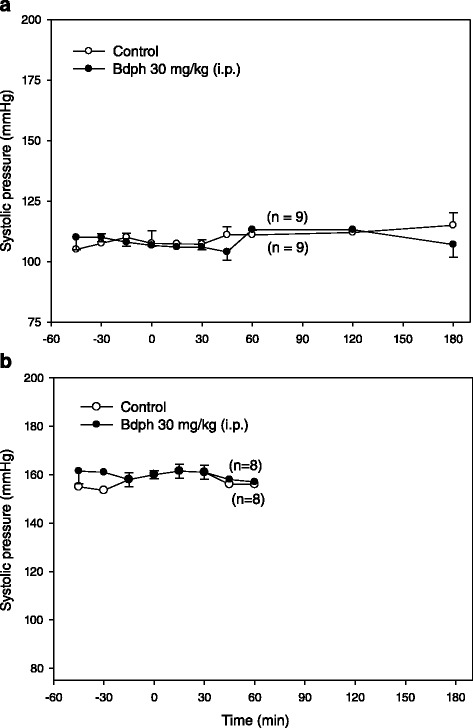
Time course of effects of butylidenephthalide (Bdph) or its vehicle (control) on systolic pressure in conscious normotensive (**a**) and spontaneous hypertensive (**b**) rats. Bdph was injected at 0 min just after the last determination of baseline values. Each point represents mean ± SEM, and n is the number of rats. There is no significant difference either between test and control (Student’s unpaired *t*-test), or between before and after Bdph injection (Student’s paired *t*-test)

The mean values of baseline systolic pressure of normotensive and spontaneous hypertensive rats were 112 ± 4 mmHg (*n* = 9) and 160 ± 2 mmHg (*n* = 8), respectively. Bdph (30 mg/kg, i.p.) had no effects on systolic pressure in normotensive and spontaneous hypertensive rats, between before and after injection or between test and control (Fig. 2a, b).

### No effects of Bdph on prazosin- and clonidine-induced systolic pressure reduction in normotensive rats

Bdph (30 mg/kg, i.p.) did not significantly affect the log dose–response curves of prazosin and clonidine when compared to their controls (data not shown).

### No effect of Bdph on Bay K 8644-induced hypertensive response in normotensive rats

Bdph (30 mg/kg, i.p.) did not significantly affect the log dose–response curve of Bay K 8644, when compared to its control (data not shown).

However, nifedipine (1 mg/kg, i.v.) downward shifted the log dose–response curve of Bay K 8644, and significantly reduced the change of systolic pressure at each dose of Bay K 8644 when compared to its control (data not shown).

### Effects of Bdph and other K^+^ channel blockers on cromakalim-induced systolic pressure reduction in normotensive rats

**Fig. 3:**
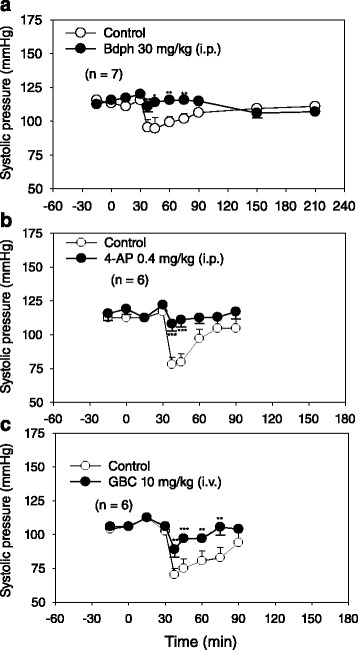
Time course of effects of cromakalim on systolic pressure in conscious normotensive rats, pretreated with butylidenephthalide (Bdph, **a**), 4-aminopyridine (4-AP, **b**), glibenclamide (GBC, **c**) or their vehicles (controls). After both second baseline values were determined, Bdph, 4-AP or GBC was immediately injected at 0 min. Thirty min after injection of Bdph, 4-AP or GBC, 0.1, 0.15, or 0.3 mg/kg of cromakalim was injected (i.v.), respectively. Each point represents mean ± SEM, and n is the number of rats. * *P* < 0.05, ** *P* < 0.01, *** *P* < 0.001 compared to their respective control

The pretreatment of Bdph (30 mg/kg, i.p.) or 4-AP (0.4 mg/kg, i.p.) did not influence the baseline systolic pressure in normotensive rats. Both treatments significantly antagonized the cromakalim-induced decrease of systolic pressure (Fig. 3a, b). The antagonistic effect of Bdph lasted for 45 min, but that of 4-AP did only for 15 min (Fig. 3a, b). The pretreatment of GBC (10 mg/kg, i.v.) also did not influence baseline systolic pressure, but significantly antagonized cromakalim-induced decrease of systolic pressure (Fig. 3c). The antagonistic effect of GBC lasted for 45 min.

**Fig. 4:**
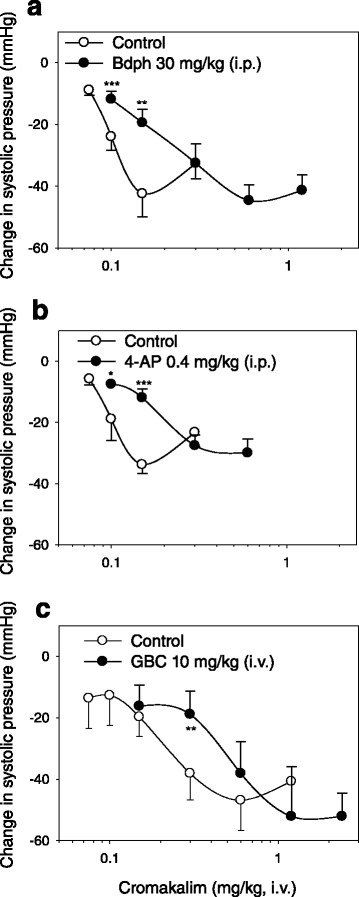
Effects of butylidenephthalide (Bdph, **a**), 4-aminopyridine (4-AP, **b**) and glibenclamide (GBC, **c**) on log dose–response curves of cromakalim for change of systolic pressure in conscious normotensive rats. All values are shown as mean ± SEM. The number of Bdph-treated rats was 8, and that of 4-AP- or GBC-treated rats was 6. * *P* < 0.05, ** *P* < 0.01 and *** *P* < 0.001 compared to their respective control

Bdph (30 mg/kg, i.p.) and 4-AP (0.4 mg/kg, i.p.) non-parallelly but surmountably rightward shifted the log-dose response curve of cromakalim for change in systolic pressure (Fig. 4a, b). Both slopes of curves were significantly greater than their controls without influencing their E_max_ values, and significantly increased their ED_50_ values, respectively (Table 1). However, GBC (10 mg/kg, i.v.) parallelly and surmountably (competitively) rightward shifted the log-dose response curve of cromakalim for change in systolic pressure (Fig. 4c), as the slope and E_max_ values were not significantly different from its control (vehicle). GBC also significantly increased the ED_50_ value (Table 1).

**Table 1:** Values of ED_50_, E_max_ and slope (S) of log dose–response curves of cromakalim in the presence and absence of antagonists, such as butylidenephthalide (Bdph), 4-aminopyridine (4-AP) and glibenclamide (GBC) in conscious normotensive rats

Response	Antagonists	ED_50_ (mg/kg)	E_max_	S	n
Change in systolic pressure (mmHg)	Bdph (30 mg/kg, i.p.)	0.25 ± 0.01^a^	−45 ± 5	−0.86 ± 0.04^a^	8
	Vehicle (1 ml/kg, i.p.)	0.11 ± 0.00	−43 ± 6	−2.16 ± 0.29	8
	4-AP (0.4 mg/kg, i.p.)	0.18 ± 0.00^a^	−30 ± 4	−0.82 ± 0.08^a^	6
	Vehicle (1 ml/kg, i.p.)	0.10 ± 0.00	−34 ± 3	−1.83 ± 0.06	6
	GBC (10 mg/kg, i.v.)	0.52 ± 0.01^a^	−53 ± 6	−1.08 ± 0.20	6
	Vehicle (1 ml/kg, i.v.)	0.23 ± 0.01	−47 ± 4	−0.72 ± 0.15	6

All values are shown as mean ± SEM, and n is the number of rats

^a^Significantly different from vehicle (*P* < 0.01)

## Discussion

Bdph at the dose of 30 mg/kg (i.p.) did not influence systolic pressure in conscious normotensive or spontaneous hypertensive rats, although Bdph at the dose was reported to reduce the systolic pressure in anesthetized renal hypertensive rats [6], suggesting that conscious rats keep intact reflex to offset the effect of Bdph on systolic pressure. Bdph at this dose did not affect the log dose–response curves of prazosin and clonidine for changes of systolic pressure in conscious normotensive rats, suggesting that Bdph at this dose did not block α_1_- or activate α_2_-adrenoceptors. In this animal model, Bdph at this dose also did not affect the log dose–response curve of Bay K 8644 for changes of systolic pressure. In contrast, nifedipine (1 mg/kg, i.v.), a reference drug, significantly reduced the change of systolic pressure at each dose of Bay K 8644 when compared to its control. Thus Bdph at this dose did not block Ca^2+^ channels in conscious normotensive rats. However, Bdph at this dose (30 mg/kg, i.p.) similar to 4-AP (0.4 mg/kg, i.p.) antagonized cromakalim-induced decrease of systolic pressure (Fig. 3a, b). Cromakalim was reported to be an ATP-sensitive K^+^ channel opener [21], which may increase outflux of K^+^ and hyperpolarize the membrane of vascular smooth muscle cells and cause decrease of systolic pressure. In the present results, GBC (10 mg/kg, i.v.), an ATP-sensitive K^+^ channel blocker [19], competitively rightward shifted the log dose–response curve of cromakalim for changes in systolic pressure. Thus, the antagonistic effect of Bdph against cromakalim was similar to that of 4-AP, a K^+^ channel blocker of K_v_1 family, and partially similar to that of GBC, an ATP-sensitive K^+^ channel blocker. Thus, Bdph may be a kind of K^+^ channel blockers, which have been reviewed to have a potential clinical use for Alzheimer disease [22]. Indeed, Bdph has also been reported to reverse the deficits of inhibitory avoidance performance and improve memory in rats [23]. However, further investigation is needed to determine Bdph how to dock in cromakalim binding sites.

GBC is one of sulfonylureas, which are powerful oral hypoglycemic drugs that have been used to treat diabetic patients for decades. Since Bdph can antagonize cromakalim-induced systolic pressure reduction in the present study, it is not surprising that Bdph was recently reported to have antihyperglycemic activity in mice [24]. Episodic ataxia type 2 (EA2) is a form of hereditary neurological disorder caused by cerebellar malfunction and is characterized by interictal ataxia and frequent attacks of dyskinesia, vertigo, and imbalance [25]. Recently, 4-AP was reported to treat EA2 [26, 27]. The target of 4-AP are K_v_1 family of K^+^ channels, possibly the K_v_1.5 subtype [28]. Further investigation is needed to determine whether Bdph is useful in treating EA2.

## Conclusions

In conclusion, the antagonistic effect of Bdph at the dose of 30 mg/kg (i.p.) on cromakalim-induced systolic pressure reduction in conscious normotensive rats was similar to that of 4-AP, a K^+^ channel blocker of K_v_1 family, and partially similar to that of GBC, a blocker of ATP-sensitive K^+^ channels. Thus Bdph may be a kind of K^+^ channel blockers.

## Abbreviations

4-AP: 4-Aminopyridine
ATP: Adenosine triphosphate
Bdph: Butylidenephthalide
GBC: Glibenclamide

## References

[1] Ko WC, Lin SC, Yeh CY, Wang YT. Alkylphthalides isolated from *Ligusticum wallichii* Franch and their in vitro inhibitory effect on rat uterine contraction induced by prostaglandin F_2α_. Taiwan Yi Xue Hui Za Zhi. 1977;76(9):669–77. 271195

[2] Ko WC, Wang YT, Lin LC. Phytochemical studies on spasmolytic constituents of *Ligusticum wallichii* Franch. Chemistry (the Cinese Chem Soc, Taiwan). 1978;67(3):74–6.

[3] Teng CM, Chen WY, Ko WC, Ouyang CH (1987). Antiplatelet effect of butylidenephthalide. Biochim Biophys Acta.

[4] Kobayashi S, Mimura Y, Notoya K, Kimura I, Kimura M (1992). Antiproliferative effects of the traditional Chinese medicine shimotsu-to, its component cnidium rhizome and derived compounds on primary cultures of mouse aorta smooth muscle cells. Jpn J Pharmacol.

[5] Kobayashi S, Mimura Y, Naitoh T, Kimura I, Kimura M (1993). Chemical structure-activity of cnidium rhizome-derived phthalides for the competence inhibition of proliferation in primary cultures of mouse aorta smooth muscle cells. Jpn J Pharmacol.

[6] Ko WC, Chang LD, Wang GY (1994). Pharmacological effects of butylidenephthalide. Phytother Res.

[7] Ko WC, Sheu JR, Tzeng SH, Chen CM (1998). The selective antianginal effect without changing blood pressure of butylidenephthalide in conscious rats. Planta Med.

[8] Ko WC, Charng CY, Sheu JR, Tzeng SH, Chen CM (1998). Effect of butylidenephthalide on calcium mobilization in isolated rat aorta. J Pharm Pharmacol.

[9] Nam KN, Kim KP, Cho KH, Jung WS, Park JM, Cho SY (2013). Prevention of inflammation-mediated neurotoxicity by butylidenephthalide and its role in microglial activation. Cell Biochem Funct.

[10] Liu SP, Harn HJ, Chien YJ, Chang CH, Hsu CY, Fu RH (2012). n-Butylidenephthalide (BP) maintains stem cell pluripotency by activating Jak2/Stat3 pathway and increases the efficiency of iPS cells generation. PLoS One.

[11] Chen YL, Jian MH, Lin CC, Kang JC, Chen SP, Lin PC (2008). The induction of orphan nuclear receptor Nur77 expression by n-butylenephthalide as pharmaceuticals on hepatocellular carcinoma cell therapy. Mol Pharmacol.

[12] Kan WL, Cho CH, Rudd JA, Lin G (2008). Study of the anti-proliferative effects and synergy of phthalides from *Angelica sinensis* on colon cancer cells. J Ethnopharmacol.

[13] Chiu SC, Chen SP, Huang SY, Wang MJ, Lin SZ, Harn HJ (2012). Induction of apoptosis coupled to endoplasmic reticulum stress in human prostate cancer cells by n-butylidenephthalide. PLoS One.

[14] Zhang H, Han T, Yu CH, Jiang YP, Peng C, Ran X (2012). Analysis of the chemical composition, acute toxicity and skin sensitivity of essential oil from rhizomes of *Ligusticum chuanxiong*. J Ethnopharmacol.

[15] Ko WC. A newly isolated antispasmodic–butylidenephthalide. Jpn J Pharmacol. 1980;30(1):85–91. 10.1254/jjp.30.85 7401411

[16] Ko WC, Lin LC, Lin SH, Hwang PY, Hsu CY, Wang GY (1992). Effects of alkylidenephthalides on the pituitrin-induced alternations in isolated guinea pig hearts. J Chin Med.

[17] Hsu HT, Yang YL, Chen WC, Chen CM, Ko WC. Butylidenephthalide blocks potassium channels and enhances basal tension in isolated guinea-pig trachea. Biomed Res Int. 2014;2014:875230. 10.1155/2014/875230 PMC4119919 25114927

[18] Mowry DT, Ringwald EL, Renoll M (1949). Vinyl aromatic compounds. VI. Alkylidenephthalides and related compounds. J Am Chem Soc.

[19] Schmid-Antomarchi H, De WJ, Fosset M, Lazdunski M (1987). The receptor for antidiabetic sulfonylureas controls the activity of the ATP-modulated K^+^ channel in insulin-secreting cells. J Biol Chem.

[20] Pfeffer JM, Pfeffer MA, Frohlich ED (1971). Validity of an indirect tail-cuff method for determining systolic arterial pressure in unanesthetized normotensive and spontaneously hypertensive rats. J Lab Clin Med.

[21] Escande D, Thuringer D, Leguern S, Cavero I (1988). The potassium channel opener cromakalim (BRL 34915) activates ATP-dependent K^+^ channels in isolated cardiac myocytes. Biochem Biophys Res Commun.

[22] Lavretsky EP, Jarvik LF (1992). A group of potassium-channel blockers-acetylcholine releasers: new potentials for Alzheimer disease?. J Clin Psychopharmacol.

[23] Hsieh MT, Wu CR, Lin LW, Hsieh CC, Tsai CH (2001). Reversal caused by n-butylidenephthalide from the deficits of inhibitory avoidance performance in rats. Planta Med.

[24] Brindis F, Rodriguez R, Bye R, Gonzalez-Andrade M, Mata R (2011). (Z)-3-butylidenephthalide from *Ligusticum porteri*, an α-glucosidase inhibitor. J Nat Prod.

[25] Jen JC, Graves TD, Hess EJ, Hanna MG, Griggs RC, Baloh RW (2007). Primary episodic ataxias: diagnosis, pathogenesis and treatment. Brain.

[26] Strupp M, Brandt T (2006). Pharmacological advances in the treatment of neuro-otological and eye movement disorders. Curr Opin Neurol.

[27] Strupp M, Kalla R, Glasauer S, Wagner J, Hufner K, Jahn K (2008). Aminopyridines for the treatment of cerebellar and ocular motor disorders. Prog Brain Res.

[28] Alvina K, Khodakhah K (2010). The therapeutic mode of action of 4-aminopyridine in cerebellar ataxia. J Neurosci.

